# Impact of Left Ventricular Mass on Mortality in Symptomatic Severe Aortic Stenosis: A Sex-Specific Analysis †

**DOI:** 10.3390/life15050814

**Published:** 2025-05-20

**Authors:** Solange Desirée Avakian, Flávio Tarasoutchi, Antonio de Padua Mansur

**Affiliations:** 1 Unidade Clinica de Valvopatias, Instituto do Coracao (InCor), Hospital das Clinicas HCFMUSP, Faculdade de Medicina, Universidade de Sao Paulo, Sao Paulo 05403-900, SP, Brazil; solange.avakian@incor.usp.br (S.D.A.); flavio.tarasoutchi@fm.usp.br (F.T.); 2 Serviço de Prevencao, Cardiopatia na Mulher e Reabilitação Cardiovascular, Instituto do Coracao (InCor), Hospital das Clinicas HCFMUSP, Faculdade de Medicina, Universidade de Sao Paulo, Sao Paulo 05403-900, SP, Brazil

**Keywords:** aortic valve stenosis, left ventricular mass, prognosis, women, men

## Abstract

Aortic stenosis (AS) is a common and serious valvular disease in older adults, often leading to increased left ventricular mass (LVM) due to pressure overload. Excessive LVM is linked to adverse outcomes, but its sex-specific prognostic significance remains unclear. Focusing on sex-based differences, this study evaluated the left ventricular mass index (LVMi) prognostic value in patients with symptomatic severe AS. We retrospectively analyzed 531 outpatients (283 men, 248 women; mean age 74.7 years) with symptomatic but stable severe AS and no prior valve procedures. Clinical and echocardiographic data were collected between April 2020 and February 2024, with a mean follow-up of 2.67 years. A total of 165 patients (31.1%) died during follow-up, 86% from cardiovascular causes. Deceased patients had lower ejection fraction and higher LVMi. Multivariate Cox analysis identified LVMi and atrial fibrillation (AF) as independent predictors of mortality, while valve intervention predicted survival. In women, both LVMi and AF predicted mortality; valve intervention was protective. In men, only the lack of valve intervention predicted death. Elevated LVMi was a strong predictor of mortality in non-operated patients, with the most pronounced impact observed in women with severe AS.

## 1. Introduction

Patients with symptomatic severe aortic stenosis (AS) face a substantially poor quality of life and an elevated risk of mortality. While traditional clinical symptoms and echocardiographic parameters have been well established as predictors of adverse outcomes [1,2,3,4], the prognostic significance of left ventricular mass (LVM) remains an area of ongoing investigation and debate. Increased LVM represents a maladaptive response to chronic pressure overload, which induces left ventricular hypertrophy, myocardial fibrosis, and compromised diastolic function [5,6]. These structural and functional alterations contribute to decreased cardiac output, heightened susceptibility to arrhythmias, and an increased risk of heart failure [7,8,9,10].

Numerous studies have reported a robust association between elevated LVM and poor clinical outcomes in patients with AS [11,12]. However, inconsistencies in the findings persist, with some studies demonstrating a significant correlation while others report a weaker or negligible association [13,14,15]. These discrepancies may be due to variations in study design, patient cohorts, and methodological approaches. Furthermore, the intricate relationship between LVM and other clinical or echocardiographic variables, such as age, hypertension, and coronary artery disease, complicates the interpretation of its role in disease progression and prognosis.

Sex constitutes a fundamental biological determinant in the development and progression of cardiovascular diseases, including AS. Women with AS frequently present with distinct clinical and echocardiographic characteristics compared to men, including older age at diagnosis, and a higher burden of comorbidities such as hypertension and diabetes [16]. Anatomically, they exhibit smaller aortic valve areas and left ventricular outflow tracts, resulting in reduced stroke volumes and an increased prevalence of paroxysmal low-flow, low-gradient AS. These features commonly accompany concentric left ventricular remodeling, typified by reduced cavity size, elevated filling pressures, lower wall stress, and more pronounced diastolic dysfunction [17]. Moreover, the prognostic impact of LVM may differ between sexes, potentially reflecting intrinsic sex-related differences in myocardial structure, function, and adaptive responses to pressure overload. Such sex-specific variations underscore the necessity of individualized assessment strategies for LVM and tailored management approaches in patients with AS. In this context, the present study aimed to investigate the prognostic relevance of LVMi in patients with symptomatic severe AS, with particular attention to sex-based differences.

## 2. Materials and Methods

This single-center retrospective observational study included 531 outpatients diagnosed with symptomatic but stable severe AS between April 2020 and February 2024. The primary objective was to evaluate the impact of LVM on mortality in patients awaiting valve intervention. Inclusion criteria required patients to have symptomatic but stable severe AS while awaiting valve intervention. Exclusion criteria included a history of prior valve procedures and the need for urgent intervention due to clinical instability. The study began as our hospital prioritized COVID-19 patients, which disrupted care for individuals requiring elective procedures, including cardiac surgeries. To reduce risks, the hospital implemented cautious strategies for interventional procedures. Elective cardiac surgeries were deferred, with priority given to patients presenting with clinically unstable AS. We followed the Strengthening the Reporting of Observational Studies in Epidemiology (STROBE) reporting guidelines.

The primary symptoms assessed were dyspnea, angina, and syncope. Dyspnea was classified according to the New York Heart Association (NYHA) functional classification, while angina was evaluated using the Canadian Cardiovascular Society (CCS) functional classification. Syncope was defined as a transient loss of consciousness followed by spontaneous recovery.

In addition to assessing clinical symptoms, the study examined the prevalence of persistent atrial fibrillation (AF) and several traditional cardiovascular risk factors, including hypertension, diabetes, dyslipidemia, smoking status, and anemia. Smokers were categorized as either current or non-smokers. Hypertension was defined as having a systolic blood pressure greater than 140 mmHg, a diastolic blood pressure above 90 mmHg, or using antihypertensive medication. Dyslipidemia was diagnosed in cases where the total cholesterol level was 240 mg/dL or higher, the triglyceride level was 150 mg/dL or higher, the low-density lipoprotein (LDL) cholesterol level exceeded 130 mg/dL, or lipid-lowering therapy was in use. Diabetes was diagnosed based on fasting glucose levels of 126 mg/dL or greater, casual plasma glucose levels of 200 mg/dL or higher, or using hypoglycemic agents. Anemia was defined as a hemoglobin plasma concentration below 13.0 g/dL for men and 12.0 g/dL for women, based on the lower cut-off of our laboratory’s normal reference values.

The diagnosis of severe AS was confirmed through a transthoracic echocardiogram conducted according to a standardized protocol at the study’s Echocardiography Core Laboratory. Several critical echocardiographic criteria determined the severity of AS: a mean transvalvular gradient of ≥40 mmHg, an aortic valve area (AVA) of ≤1.0 cm^2^, or a peak aortic jet velocity of ≥4.0 m per second. We calculated LVEF as the ratio of stroke volume to end-diastolic volume. LV volumes were determined using the biplane disc method, a modified version of Simpson’s rule. This method involves summing the volumes of small cylindrical discs to estimate the total LV volume.

The LVMi was calculated by dividing the LVM by the body surface area (BSA). LVM was derived using the following formula [18]:

LVM (grams) = 0.8 × 1.04 × {[(LV diastolic diameter + diastolic interventricular septal thickness + LV posterior wall thickness in diastole)3 − LV diastolic diameter3]} + 0.6

Reference values for LVMi were based on guidelines from the American Society of Echocardiography, which defined left ventricular hypertrophy as an LVMi greater than 95 g/m^2^ for women and 115 g/m^2^ for men [19].

The study assessed various valve interventions, including aortic valve replacement (AVR), valvuloplasty, and transcatheter aortic valve implantation (TAVI). As part of the follow-up protocol, we recorded whether each patient underwent any of these procedures during the study period, categorizing the variable “valve intervention” as either “yes” or “no”. Additionally, we evaluated the prevalence of coronary artery disease (CAD), defined as the presence of at least one major subepicardial coronary artery with a ≥70% stenotic lesion.

### Statistical Analysis

Statistical analysis was conducted by expressing continuous variables as means with standard deviations and categorical variables as frequencies with corresponding percentages. The assumption of normality was assessed using tests for equality of variances. Continuous variables were compared between groups using Student’s *t*-test, while categorical variables were compared using the chi-square test. A *p*-value of less than 0.05 was considered statistically significant. The cumulative incidence of all-cause mortality was estimated using the Kaplan–Meier (K-M) method, incorporating Šidák adjustments for multiple comparisons. LVMi was categorized into two strata: stratum 1, which includes normal (43–95 g/m^2^ for women and 49–115 g/m^2^ for men) to mildly abnormal LVMi (96–108 g/m^2^ for women and 116–131 g/m^2^ for men), and stratum 2, which includes moderate (109–121 g/m^2^ for women and 132–148 g/m^2^ for men) to severely abnormal LVMi (≥122 g/m^2^ for women and ≥149 g/m^2^ for men) [19]. Cox proportional hazard models were utilized to identify variables independently associated with all-cause mortality. The chi-square score statistic from the Cox model was used to identify the most significant predictors of mortality. The dependent variable in these models was death, with adjustments made for covariates exhibiting a *p*-value less than 0.1, including LVEF, LVMi, CAD, angina, AF, and valve interventions that included AVR, TAVI, or valvuloplasty. Separate Cox proportional hazard models were constructed for male and female patients, using the same covariates in each model. All statistical analyses were performed using the SAS^®^ Studio software package (Version 3.8) (SAS Institute, Cary, NC, USA).

## 3. Results

The analysis included a cohort of 531 patients, with a mean age of 74.7 ± 11.6 years, of which 253 (53.3%) were male. Women exhibited a higher prevalence of dyslipidemia, hypertension, higher LVEF, moderate to severely abnormal LVMi, left ventricular (LV) gradient, and peak aortic jet velocity. Conversely, men had a higher prevalence of AF, larger BSA, higher LVMi, and a higher incidence of CAD. AVR was performed in 91 men (32%) and 71 women (29%) (*p* = 0.378), while TAVI was conducted in 46 men (16%) and 52 women (21%) (*p* = 0.163) (Table 1). Among those who underwent AVR (n = 162; 31% of the cohort), the primary indications included clinical decompensation, CCS class III/IV angina in 75 patients (46%), and NYHA class III/IV dyspnea in 45 patients (28%). TAVI was performed in 98 patients (19%), with CCS class III/IV angina and NYHA class III/IV dyspnea as the primary indication in 39 (40%) and 36 (37%) patients, respectively. The remaining AVR and TAVI procedures were carried out electively in stable patients through routine evaluation by the Valve Unit without evidence of clinical deterioration.

Survivors were more likely to have angina, higher LVEF, and to have undergone AVR or TAVI. In contrast, non-survivors had a higher prevalence of AF, elevated LVMi, and moderate to severely abnormal LVMi and were more likely to have undergone valvuloplasty (Table 2).

Among survivors, women had higher rates of hypertension, LVEF, moderate to severely abnormal LVMi, LV gradient, and peak aortic velocity. In contrast, men had a higher prevalence of syncope, AF, larger BSA, higher LVMi, and CAD. During the study period of 2.7 ± 1.2 years, 165 patients (31.1%) died, with 148 (90%) of these deaths attributed to cardiovascular disease, 79 (53.9%) in men and 69 (46.1%) in women. There were seventeen non-cardiovascular deaths (10%), including eight (47.1%) from COVID-19 (six men, two women), seven (41.2%) from cancer (two men, five women), and two from sepsis (both in men). Among the non-survivors, women had higher rates of dyslipidemia, elevated LVEF, moderate to severely abnormal LVMi, higher LV gradient, peak aortic velocity, and lower valve area, while men demonstrated a higher prevalence of diabetes, larger BSA, larger aortic valve area, and CAD. Women in the non-survivor group exhibited a significantly higher prevalence of moderate to severely abnormal LVMi compared to men. LVEF was nearly significantly higher in both surviving women (*p* = 0.056) and surviving men (*p* = 0.052) compared to non-surviving women and men, respectively (Table 3).

**Figure 1:**
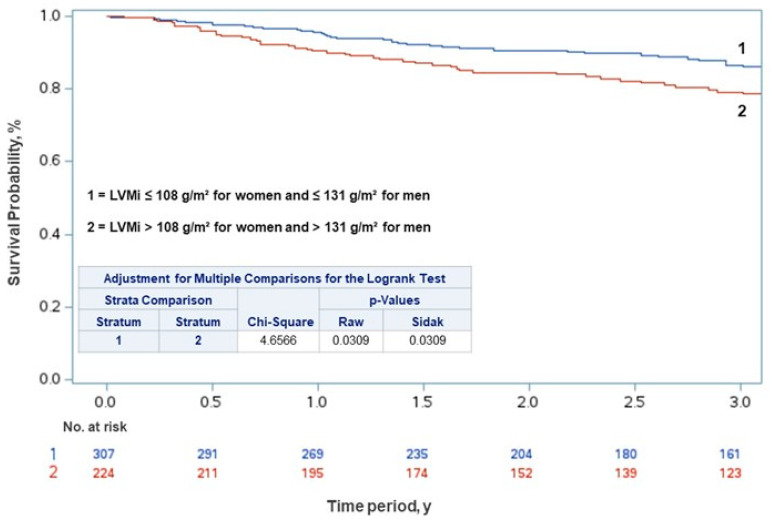
Kaplan–Meier curves showing the risk of death by left ventricular mass index (LVMi) in symptomatic severe aortic stenosis.

Kaplan–Meier survival analysis showed a higher cumulative incidence of death in patients with moderate to severely abnormal LVMi compared to patients with normal to slightly abnormal LVMi (stratum 1 vs. 2; *p* = 0.031) (Figure 1).

**Figure 2:**
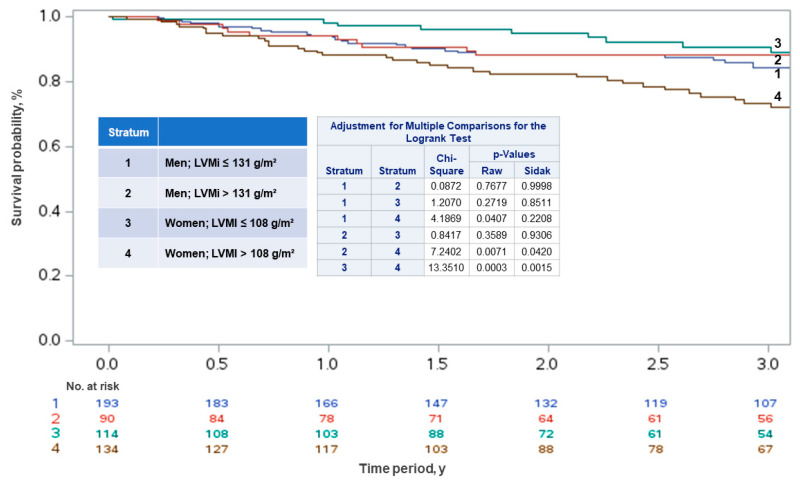
Kaplan–Meier curves showing risk of death based on left ventricular mass index (LVMi) in women and men with symptomatic severe aortic stenosis.

A cumulative incidence of death was also higher in women with moderate to severely abnormal LVMi compared to men with the same classification (stratum 4 vs. 2; *p* = 0.042). Additionally, women with moderate to severely abnormal LVMi had a significantly higher incidence of death compared to women with normal to slightly abnormal LVMi (stratum 4 vs. 3; *p* = 0.002) (Figure 2).

**Figure 3:**
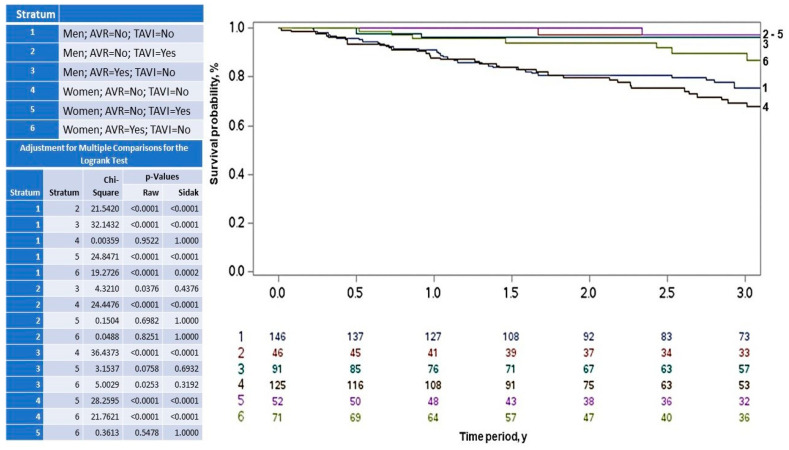
Kaplan–Meier curves showing the risk of death in women and men with symptomatic severe aortic stenosis who underwent surgical aortic valve replacement (AVR) or transcatheter aortic valve implantation (TAVI).

Among the 258 patients who did not undergo any intervention, 124 (48%) died, 66 men (53%) and 58 women (47%), with no significant sex-based difference (*p* = 0.873). As shown in Table 3, postoperative death occurred in 20 patients (3.8%) who underwent aortic valve replacement (AVR) and in 13 patients (2.5%) who underwent transcatheter aortic valve implantation (TAVI), with no significant difference between the two interventions (*p* = 0.901). Mortality rates also did not differ significantly by sex within each intervention. Among those who underwent AVR, nine men (3.2%) and 11 women (4.4%) died. In the TAVI group, eight men (2.8%) and five women (2.0%) died (*p* = 0.567). After Šidák adjustment for multiple comparisons, the cumulative incidence of death remained similar between women and men who underwent AVR (strata 3 vs. 6; *p* = 0.319) and TAVI (strata 2 vs. 5; *p* = 1.0). Likewise, no significant differences were observed between women who underwent AVR vs. TAVI (strata 5 vs. 6; *p* = 1.0) or men who underwent AVR vs. TAVI (strata 2 vs. 3; *p* = 0.438) (Figure 3).

Cox multivariate analysis identified LVMi [HR = 1.39 (95% CI: 1.02–1.90); *p* = 0.032] and AF [HR = 1.68 (95% CI: 1.02–2.74); *p* = 0.038] as independent predictors of mortality, while valve intervention [HR = 0.47 (95% CI: 0.37–0.61); *p* < 0.001] was the only independent predictor of survival in the overall cohort. In women, both LVMi [HR = 2.52 (95% CI: 1.48–4.28); *p* = 0.001] and AF [HR = 2.77 (95% CI: 1.18–6.50); *p* = 0.014] were independent predictors of mortality, while valve intervention [HR = 0.43 (95% CI: 0.30–0.63); *p* < 0.001] significantly predicted survival. In men, after adjusting for the same covariates, only valve intervention [HR = 0.51 (95% CI: 0.37–0.71); *p* < 0.001] significantly predicted survival, with no other factors independently associated with mortality. Chi-square test scores further supported these findings: in the overall cohort, LVMi (χ^2^ = 4.6) and AF (χ^2^ = 4.3) were the strongest predictors of death, while valve intervention (χ^2^ = 40.3) was the sole predictor of survival. Among women, LVMi (χ^2^ = 11.7) was the most robust mortality predictor, followed by AF (χ^2^ = 6.0), with valve intervention (χ^2^ = 23.3) as the primary survival predictor. In men, only valve intervention (χ^2^ = 23.3) predicted survival.

## 4. Discussion

Our study identified a strong, statistically significant correlation between LVMi and mortality in women with symptomatic severe aortic stenosis (AS), establishing LVMi as the most robust predictor of death in this group. These findings align with previous research [20,21], including the PARTNER Trials and Registries [22], which demonstrated that elevated LVMi is independently associated with all-cause and cardiovascular mortality, even after adjusting for multiple covariates. Consistent with our results, earlier studies also reported that LVMi serves as an independent predictor of all-cause and cardiovascular death in women but not in men [14,23,24].

LVMi is a well-established prognostic marker, with higher values strongly linked to an increased risk of cardiovascular events in patients with AS. Its consistent association with adverse outcomes highlights its importance as a critical indicator of prognosis, particularly in women with severe AS.

In our cohort, men exhibited higher overall LVMi values. However, when applying sex-specific thresholds for moderate and severe LVMi, women showed a higher prevalence of elevated LVMi. This observation is in line with previous studies [25,26,27]. Despite these findings, differences in prognosis and optimal mortality predictors between women and men with symptomatic severe AS awaiting surgical intervention remain insufficiently explored.

It is well established that women with moderate to severe LVMi face a worse prognosis than men with similar LVMi levels. Several factors may explain this sex-based disparity. Women often exhibit more pronounced concentric remodeling and hypertrophy than men [28], characterized by higher relative wall thickness and smaller LV dimensions. This concentric hypertrophy pattern has a significantly greater impact on prognosis in women. Additionally, outcomes following AVR show that residual LVH is associated with increased mortality, with a stronger effect observed in women. This suggests that women are more adversely affected by residual LVH than men [29]. Women may also develop a more intense form of concentric remodeling and hypertrophy in response to pressure overload, resulting in greater LV cavity size reduction, decreased compliance, impaired filling and pump function, and lower stroke volume than men [30].

Furthermore, women may be more prone to developing diffuse myocardial fibrosis, which can be detected by cardiac magnetic resonance imaging regardless of AS severity [31]. However, there is ongoing debate about whether diffuse myocardial fibrosis is more prevalent in women, and the mechanisms underlying sex-related differences in LV remodeling and fibrosis remain unclear [32,33,34].

A recent study of patients with severe AS undergoing AVR found that women had less replacement fibrosis than men. Despite this, while women showed lower overall mortality, replacement fibrosis in both sexes was associated with LV decompensation and increased post-AVR mortality. These findings suggest that, even with lower fibrosis levels in women, the extent of fibrosis remains a critical factor influencing mortality in both sexes [35].

The mechanism underlying sex differences in myocardial fibrosis remains unclear. Sex hormones, particularly estrogen and testosterone, play influential roles, with estrogen offering cardioprotective effects in women and testosterone promoting hypertrophy and fibrosis in men [36,37]. However, because most patients with AS are over 60, the role of estrogen in postmenopausal women is not fully understood [38]. Estrogen loss after menopause may increase susceptibility to LV wall stress, myocardial ischemia, increased LVM, and fibrosis, which tend to worsen with age in women but not in men [39,40].

Studies suggest that estrogen downregulates the expression of collagen I and III genes in female—but not male—cardiac fibroblasts [41], thereby inhibiting fibroblast proliferation and reducing collagen protein synthesis [42]. Despite this, the exact role of estrogen in the fibrotic process among postmenopausal women with AS remains uncertain and requires further investigation. The absence of estrogen may increase vulnerability to LV wall stress and ischemia, a hypothesis that merits deeper study.

In patients with concentric LVH, several factors reduce coronary blood flow, including decreased perfusion pressure, increased external compressive forces, shortened diastolic perfusion time, and reduced microvascular density [43,44]. Elevated intracavitary pressure exacerbates these effects, increasing the risk of myocardial ischemia and related complications due to impaired perfusion.

These changes can reverse the typical endocardial-to-epicardial blood flow gradient, significantly reducing subendocardial perfusion [45]. This shift is central to AS pathophysiology and contributes to subendocardial ischemia, cell death, and fibrosis.

Our study also identified AF as an independent predictor of higher mortality, consistent with previous literature [46]. AF is a well-known risk factor for poor outcomes due to its association with complications such as stroke and heart failure. The alignment of our findings with established research highlights the importance of monitoring and managing AF and related cardiovascular risks to improve patient outcomes.

This study has several limitations. As a single-center, retrospective observational analysis, its generalizability is limited, especially in settings with different patient demographics, clinical practices, and treatment protocols. The study was conducted during the COVID-19 pandemic, when elective surgical interventions were not performed, which may have significantly influenced outcomes. Notably, most adverse outcomes occurred among patients who had indications for surgery but did not undergo intervention, indicating that the observed prognostic impact of LVMi is specific to the non-operated cohort. Echocardiography, while routinely used to assess LVMi in patients with aortic valve stenosis, is less accurate than advanced imaging modalities such as magnetic resonance imaging or computed tomography, potentially introducing measurement variability. In addition, key echocardiographic parameters, including pulmonary artery systolic pressure and the severity of diastolic dysfunction, were not consistently available, limiting a comprehensive evaluation of cardiovascular status. The study also did not include invasive or non-invasive assessments of microvascular dysfunction, precluding a more detailed analysis of myocardial ischemia with or without obstructive coronary disease. Lastly, as this was an observational study, the possibility of residual confounding cannot be entirely excluded despite statistical adjustment for relevant variables. Nonetheless, the strength of this study lies in the analysis of a large, consecutive cohort of patients managed under consistent protocols, which reduces variability and reveals meaningful prognostic patterns.

## 5. Conclusions

Cox multivariate analysis revealed that LVMi and AF are independent predictors of mortality. Particularly in women, LVMi, AF, and less valve intervention were significantly associated with an increased risk of death. In contrast, less valve intervention was an independent predictor of mortality identified in men. These findings indicate that elevated LVMi serves as a critical predictor of mortality, particularly in the female population, highlighting the necessity for targeted interventions to address this demographic’s unique risks. Importantly, most of the fatal outcomes occurred in patients who were indicated for planned surgical intervention but did not undergo the procedure due to the COVID-19 pandemic. Therefore, the observed prognostic impact of LVMi is specific to the non-operated cohort, reinforcing the clinical imperative of timely surgical management when indicated.

## Figures and Tables

**Table 1:** Clinical characteristics, echocardiographic data, and surgical interventions of all patients, women, and men with symptomatic severe aortic valve stenosis.

	All Patients N = 531 (%)	Men N = 283 (53.3)	Women N = 248 (46.7)	*p*
Age, mean (SD), y	74.7 (11.6)	74.1 (12.6)	75.5 (10.2)	0.149
Time since baseline, mean (SD), y	2.7 (1.2)	2.8 (1.3)	2.6 (1.2)	0.138
Dyslipidemia, No. (%)	177 (33.3)	81 (28.6)	96 (38.7)	0.014
Hypertension, No. (%)	390 (73.6)	192 (68.1)	198 (79.8)	0.002
Diabetes, No. (%)	153 (28.8)	85 (30.0)	68 (27.4)	0.507
Smoking, No. (%)	43 (8.1)	23 (8.1)	20 (8.1)	0.979
Anemia, No. (%)	145 (27.3)	81 (28.6)	64 (25.8)	0.468
Syncope, No. (%)	46 (8.7)	32 (11.3)	14 (5.7)	0.021
Angina, No. (%)	197 (37.1)	112 (39.6)	85 (34.3)	0.207
Dyspnea, No. (%)	475 (89.5)	247 (87.3)	228 (91.9)	0.081
AF, No. (%)	33 (6.2)	24 (8.5)	9 (3.6)	0.021
Weight, mean (SD), Kg	74.3 (14.3)	79.1 (13.0)	68.6 (13.6)	<0.001
BSA, mean (SD), m^2^	1.8 (0.2)	1.9 (0.2)	1.7 (0.2)	<0.001
LVEF, mean (SD), %	60.6 (9.6)	58.7 (10.7)	62.8 (7.6)	<0.001
LVMi, mean (SD), g/m^2^	118.6 (30.6)	122.1 (29.9)	114.5 (30.9)	0.004
LVMi moderate + severe	224 (42.2)	90 (31.8)	134 (54.0)	<0.001
LA volume, mean (SD), mL	44.9 (15.2)	45.3 (14.5)	44.5 (16.0)	0.666
Peak gradient, mean (SD), mmHg	79.8 (22.9)	76.9 (21.4)	83.1 (24.2)	0.002
Mean gradient, mean (SD), mmHg	50.5 (15.6)	48.6 (14.7)	52.6 (16.3)	0.003
Valve area, mean (SD), cm^2^	0.87 (2.42)	1.0 (3.3)	0.72 (0.19)	0.155
Peak jet velocity, mean (SD), m/s	4.4 (0.6)	4.3 (0.6)	4.5 (0.6)	0.002
Bicuspid/Tricuspid, No. (%)	54 (10.2)/477 (89.8)	30 (10.6)/253 (89.4)	24 (9.68)/224 (90.3)	0.725
CAD, No. (%)	279 (52.5)	172 (60.78)	107 (43.2)	<0.001
AVR, No. (%)	162 (30.5)	91 (32.2)	71 (28.6)	0.378
Valvuloplasty, No. (%)	13 (2.45)	10 (3.5)	3 (1.21)	0.084
TAVI, No. (%)	98 (18.5)	46 (16.3)	52 (21.0)	0.163
Death, No. (%)	165 (31.1)	89 (31.5)	76 (30.7)	0.842
Cardiovascular death, No. (%)	148 (27.9)	79 (27.9)	69 (27.8)	0.921

Abbreviations: AF, atrial fibrillation; AVR, aortic valve replacement; BSA, body surface area; CAD, coronary artery disease; LA, left atrium; LVEF, left ventricular ejection fraction; LVMi, left ventricular mass index; TAVI, transcatheter aortic valve implantation.

**Table 2:** Clinical characteristics, echocardiographic data, and surgical interventions in patients with symptomatic severe aortic valve stenosis: a comparative analysis of survivors and non-survivors.

	All Patients N = 531 (%)	Survivors N = 366 (68.9)	Non-Survivors N = 165 (31.1)	*p*
Age, mean (SD), y	74.7 (11.6)	74.4 (11.3)	75.5 (12.1)	0.133
Female, No. (%)	366 (68.9)	172 (47.0)	76 (46.1)	0.841
Time since baseline, mean (SD), y	2.7 (1.2)	2.73 (1.2)	2.55 (1.3)	0.146
Dyslipidemia, No. (%)	177 (33.3)	124 (33.4)	53 (32.1)	0.691
Hypertension, No. (%)	390 (73.6)	270 (73.8)	120 (73.2)	0.885
Diabetes, No. (%)	153 (28.8)	101 (27.6)	52 (31.5)	0.356
Smoking, No. (%)	43 (8.1)	26 (7.1)	17 (10.3)	0.211
Anemia, No. (%)	145 (27.3)	96 (26.2)	49 (29.7)	0.410
Syncope, No. (%)	46 (8.7)	35 (9.6)	11 (6.7)	0.272
Angina, No. (%)	197 (37.1)	146 (39.9)	51 (30.9)	0.047
Dyspnea, No. (%)	475 (89.5)	325 (88.8)	150 (90.9)	0.463
AF, No. (%)	33 (6.2)	15 (4.1)	18 (10.9)	0.003
Weight, mean (SD), Kg	74.3 (14.3)	74.7 (14.2)	73.4 (14.6)	0.334
BSA, mean (SD), m^2^	1.8 (0.21)	1.8 (0.2)	1.7 (0.2)	0.205
LVEF, mean (SD), %	60.6 (9.6)	61.4 (8.9)	58.8 (10.8)	0.008
LVMi, mean (SD), g/m^2^	118.6 (30.6)	115.5 (29.3)	125.3 (32.5)	<0.001
LVMi moderate + severe	224 (42.2)	136 (37.2)	88 (53.3)	<0.001
LA volume, mean (SD), mL	44.9 (15.2)	44.4 (16.1)	46.3 (12.8)	0.246
Peak gradient, mean (SD), mmHg	79.8 (22.9)	80.4 (23.3)	78.4 (22.0)	0.365
Mean gradient, mean (SD), mmHg	50.5 (15.6)	50.6 (15.7)	50.4 (15.3)	0.894
Valve area, mean (SD), cm^2^	0.87 (2.42)	0.9 (2.9)	0.7 (0.2)	0.258
Peak jet velocity, mean (SD), m/s	4.4 (0.6)	4.4 (0.6)	4.4 (0.6)	0.359
Bicuspid/Tricuspid, No. (%)	54 (10.2)/477 (89.8)	42 (11.5)/324 (88.5)	12 (7.2)/153 (92.7)	0.138
CAD, No. (%)	279 (52.5)	183 (50.0)	96 (58.1)	0.081
AVR, No. (%)	162 (30.5)	142 (38.8)	20 (12.1)	<0.001
Valvuloplasty, No. (%)	13 (2.45)	5 (1.37)	8 (4.9)	0.016
TAVI, No. (%)	98 (18.5)	85 (23.2)	13 (7.9)	<0.001

Abbreviations: AF, atrial fibrillation; AVR, aortic valve replacement; BSA, body surface area; CAD, coronary artery disease; LA, left atrium; LVEF, left ventricular ejection fraction; LVMi, left ventricular mass index; TAVI, transcatheter aortic valve implantation.

**Table 3:** Clinical characteristics, echocardiographic data, and surgical interventions in women and men with symptomatic severe aortic valve stenosis: a comparative analysis of survivors and non-survivors.

	Survivors		*p*	Non-Survivors		*p*
	Men N = 194 (53)	Women N = 172 (47)		Men N = 89 (53.9)	Women N = 76 (46.1)	
Age, mean (SD), y	75.1 (10.5)	75.1 (10.8)	0.792	73.9 (15.0)	73.0 (11.2)	0.650
Time since baseline, mean (SD), y	2.8 (1.2)	2.7 (1.1)	0.284	2.7 (1.4)	2.4 (1.3)	0.289
Dyslipidemia, No. (%)	59 (30.4)	65 (37.8)	0.137	22 (24.7)	31 (40.8)	0.028
Hypertension, No. (%)	127 (65.5)	143 (83.1)	<0.001	65 (73.9)	55 (72.4)	0.829
Diabetes, No. (%)	50 (25.8)	51 (29.7)	0.408	35 (39.3)	17 (22.4)	0.019
Smoking, No. (%)	16 (8.3)	10 (5.8)	0.366	7 (7.9)	10 (13.2)	0.265
Anemia, No. (%)	52 (26.8)	44 (25.6)	0.791	29 (32.6)	20 (26.3)	0.380
Syncope, No. (%)	25 (12.9)	10 (5.8)	0.022	7 (7.9)	4 (5.3)	0.506
Angina, No. (%)	86 (44.3)	60 (34.8)	0.066	26 (29.2)	25 (32.9)	0.610
Dyspnea, No. (%)	169 (87.1)	156 (90.7)	0.279	78 (87.6)	72 (94.7)	0.114
AF, No. (%)	12 (6.19)	3 (1.74)	0.032	12 (13.5)	6 (7.9)	0.251
Weight, mean (SD), Kg	79.7 (12.6)	69.0 (13.7)	<0.001	27.9 (13.9)	67.6 (13.5)	<0.001
BSA, mean (SD), m^2^	1.9 (0.2)	1.7 (0.2)	<0.001	1.9 (0.2)	1.7 (0.2)	<0.001
LVEF, mean (SD), %	59.6 (9.7)	63.4 (6.9)	<0.001	56.7 (12.0)	61.3 (8.7)	0.006
LVMi, mean (SD), g/m^2^	120.3 (29.1)	110.1 (28.5)	<0.001	125.9 (31.5)	124.5 (33.8) ^a^	0.784
LVMi moderate + severe	60 (30.9)	76 (44.2)	0.009	30 (33.7)	58 (76.3) ^a^	<0.001
LA volume, mean (SD), mL	44.8 (15.4)	43.8 (16.9)	0.619	46.6 (12.2)	46.3 (13.7)	0.995
Peak gradient, mean (SD), mmHg	77.6 (20.5)	83.6 (25.8)	0.015	75.4 (23.1)	82.0 (20.2)	0.057
Mean gradient, mean (SD), mmHg	48.9 (14.0)	52.5 (17.3)	0.030	48.1 (16.1)	53.1 (13.9)	0.038
Valve area, mean (SD), cm^2^	1.1 (4.0)	0.7 (0.2)	0.200	0.8 (1.2)	0.7 (0.2)	<0.001
Peak jet velocity, mean (SD), m/s	4.4 (0.6)	4.5 (0.7)	0.023	4.3 (0.7)	4.5 (0.6)	0.039
Bicuspid/Tricuspid, No. (%)	23 (11.9)/171 (88.1)	19 (11.1)/153 (88.9)	0.809	7 (7.9)/82 (92.1)	5 (6.6)/71 (93.4)	0.751
CAD, No. (%)	114 (58.8)	69 (40.1)	<0.001	58 (65.2)	38 (50)	0.049
AVR, No. (%)	82 (42.3)	60 (34.9)	0.148	9 (10.1)	11 (14.5)	0.392
Valvuloplasty, No. (%)	4 (2.1)	1 (0.6)	0.223	6 (6.7)	2 (2.6)	0.220
TAVI, No. (%)	38 (19.6)	47 (27.3)	0.081	8 (9.0)	5 (6.6)	0.567

^a^ *p* < 0.001, survivors (women) vs. non-survivors (women). Abbreviations: AF, atrial fibrillation; AVR, aortic valve replacement; BSA, body surface area; CAD, coronary artery disease; LA, left atrium; LVEF, left ventricular ejection fraction; LVMi, left ventricular mass index; TAVI, transcatheter aortic valve implantation.

## Data Availability

The raw data in this article will be made available upon reasonable request to the corresponding author.

## Abbreviations

The following abbreviations are used in this manuscript:

AS	Aortic stenosis
LVM	Left ventricular mass
LVMi	Left ventricular mass index
LVEF	Left ventricular ejection fraction
AF	Atrial fibrillation
AVA	Aortic valve area
LVH	Left ventricular hypertrophy
AVR	Aortic valve replacement
TAVI	Transcatheter aortic valve implantation
CAD	Coronary artery disease
PASP	Pulmonary artery systolic pressure

## References

[1] Otto C.M., Burwash I.G., Legget M.E., Munt B.I., Fujioka M., Healy N.L., Kraft C.D., Miyake-Hull C.Y., Schwaegler R.G. (1997). Prospective study of asymptomatic valvular aortic stenosis. Clinical, echocardiographic, and exercise predictors of outcome. Circulation.

[2] Rosenhek R., Binder T., Porenta G., Lang I., Christ G., Schemper M., Maurer G., Baumgartner H. (2000). Predictors of outcome in severe, asymptomatic aortic stenosis. N. Engl. J. Med..

[3] Pellikka P.A., Sarano M.E., Nishimura R.A., Malouf J.F., Bailey K.R., Scott C.G., Barnes M.E., Tajik A.J. (2005). Outcome of 622 adults with asymptomatic, hemodynamically significant aortic stenosis during prolonged follow-up. Circulation.

[4] Avakian S.D., Grinberg M., Ramires J.A., Mansur A.P. (2008). Outcome of adults with asymptomatic severe aortic stenosis. Int. J. Cardiol..

[5] Bing R., Cavalcante J.L., Everett R.J., Clavel M.-A., Newby D.E., Dweck M.R. (2019). Imaging and impact of myocardial fibrosis in aortic stenosis. JACC Cardiovasc. Imaging.

[6] Dweck M.R., Boon N.A., Newby D.E. (2012). Calcific aortic stenosis: A disease of the valve and the myocardium. J. Am. Coll. Cardiol..

[7] De Biase N., Mazzola M., Del Punta L., Di Fiore V., De Carlo M., Giannini C., Costa G., Paneni F., Mengozzi A., Nesti L. (2023). Haemodynamic and metabolic phenotyping of patients with aortic stenosis and preserved ejection fraction: A specific phenotype of heart failure with preserved ejection fraction?. Eur. J. Heart Fail..

[8] Azevedo C.F., Nigri M., Higuchi M.L., Pomerantzeff P.M., Spina G.S., Sampaio R.O., Tarasoutchi F., Grinberg M., Rochitte C.E. (2010). Prognostic significance of myocardial fibrosis quantification by histopathology and magnetic resonance imaging in patients with severe aortic valve disease. J. Am. Coll. Cardiol..

[9] Cioffi G., Faggiano P., Vizzardi E., Tarantini L., Cramariuc D., Gerdts E., de Simone G. (2011). Prognostic effect of inappropriately high left ventricular mass in asymptomatic severe aortic stenosis. Heart.

[10] Stassen J., Ewe S.H., Hirasawa K., Butcher S.C., Singh G.K., Amanullah M.R., Sin K.Y.K., Ding Z.P., Pio S.M., Chew N.W.S. (2022). Left ventricular remodelling patterns in patients with moderate aortic stenosis. Eur. Heart J. Cardiovasc. Imaging.

[11] Lorell B.H., Carabello B.A. (2000). Left ventricular hypertrophy: Pathogenesis, detection, and prognosis. Circulation.

[12] Duncan A.I., Lowe B.S., Garcia M.J., Xu M., Gillinov A.M., Mihaljevic T., Koch C.G. (2008). Influence of concentric left ventricular remodeling on early mortality after aortic valve replacement. Ann. Thorac. Surg..

[13] Burchfield J.S., Xie M., Hill J.A. (2013). Pathological ventricular remodeling: Mechanisms: Part 1 of 2. Circulation.

[14] Minamino-Muta E., Kato T., Morimoto T., Taniguchi T., Inoko M., Haruna T., Izumi T., Miyamoto S., Nakane E., Sasaki K. (2017). Impact of the left ventricular mass index on the outcomes of severe aortic stenosis. Heart.

[15] Ito N., Zen K., Takahara M., Tani R., Nakamura S., Fujimoto T., Takamatsu K., Yashige M., Kadoya Y., Yamano M. (2023). Left ventricular hypertrophy as a predictor of cardiovascular outcomes after transcatheter aortic valve replacement. ESC Heart Fail..

[16] DesJardin J.T., Chikwe J., Hahn R.T., Hung J.W., Delling F.N. (2022). Sex Differences and Similarities in Valvular Heart Disease. Circ. Res..

[17] Ong J.Y.S., Leow A.S.T., Ng C.Y., Loh P.H., Quek S.C., Kong W.K.F., Yeo T.C., Sia C.H., Poh K.K. (2025). Longitudinal Outcomes of Patients with Aortic Stenosis Stratified by Sex: An Asian Perspective. J. Cardiovasc. Dev. Dis..

[18] Devereux R.B., Alonso D.R., Lutas E.M., Gottlieb G.J., Campo E., Sachs I., Reichek N. (1986). Echocardiographic assessment of left ventricular hypertrophy: Comparison to necropsy findings. Am. J. Cardiol..

[19] Lang R.M., Biering M., Devereux R.B., Flachskampf F.A., Foster E., Pellikka P.A., Picard M.H., Roman M.J., Seward J., Shanewise J.S. (2005). Recommendations for chamber quantification: A report from the American Society of Echocardiography’s Guidelines and Standards Committee and the Chamber Quantification Writing Group, developed in conjunction with the European Association of Echocardiography, a branch of the European Society of Cardiology. J. Am. Soc. Echocardiogr..

[20] Fuster R.G., Argudo J.A., Albarova O.G., Sos F.H., López S.C., Sorlıí M.J.D., Codoñer M.B., Miñano J.A. (2003). Left ventricular mass index in aortic valve surgery: A new index for early valve replacement?. Eur. J. Cardiothorac. Surg..

[21] Stassen J., Pio S.M., Ewe S.H., Amanullah M.R., Hirasawa K., Butcher S.C., Singh G.K., Sin K.Y., Ding Z.P., Chew N.W. (2022). Sex-Related Differences in Medically Treated Moderate Aortic Stenosis. Struct. Heart.

[22] Gonzales H., Douglas P.S., Pibarot P., Hahn R.T., Khalique O.K., Jaber W.A., Cremer P., Weissman N.J., Asch F.M., Zhang Y. (2020). Left Ventricular Hypertrophy and Clinical Outcomes Over 5 Years After TAVR: An Analysis of the PARTNER Trials and Registries. JACC Cardiovasc. Interv..

[23] Rader F., Sachdev E., Arsanjani R., Siegel R.J. (2015). Left ventricular hypertrophy in valvular aortic stenosis: Mechanisms and clinical implications. Am. J. Med..

[24] Gerdts E., Rossebø A.B., Pedersen T.R., Cioffi G., Lønnebakken M.T., Cramariuc D., Rogge B.P., Devereux R.B. (2015). Relation of Left Ventricular Mass to Prognosis in Initially Asymptomatic Mild to Moderate Aortic Valve Stenosis. Circ. Cardiovasc. Imaging.

[25] Douglas P.S., Otto C.M., Mickel M.C., Labovitz A., Reid C.L., Davis K.B. (1995). Gender differences in left ventricle geometry and function in patients undergoing balloon dilatation of the aortic valve for isolated aortic stenosis. NHLBI Balloon Valvuloplasty Registry. Br. Heart J..

[26] Pighi M., Piazza N., Martucci G., Lachapelle K., Perrault L.P., Asgar A.W., Lauck S., Webb J.G., Popma J.J., Kim D.H. (2019). Sex-Specific Determinants of Outcomes After Transcatheter Aortic Valve Replacement. Circ. Cardiovasc. Qual. Outcomes.

[27] Singh A., Musa T.A., Treibel T.A., Vassiliou V.S., Captur G., Chin C., Dobson L.E., Pica S., Loudon M., Malley T. (2019). Sex differences in left ventricular remodelling, myocardial fibrosis and mortality after aortic valve replacement. Heart.

[28] Capoulade R., Clavel M.A., Le Ven F., Dahou A., Thébault C., Tastet L., Shen M., Arsenault M., Bédard É., Beaudoin J. (2017). Impact of left ventricular remodelling patterns on outcomes in patients with aortic stenosis. Eur. Heart J. Cardiovasc. Imaging.

[29] Gavina C., Falcao-Pires I., Pinho P., Manso M.-C., Gonçalves A., Rocha-Gonçalves F., Leite-Moreira A. (2016). Relevance of residual left ventricular hypertrophy after surgery for isolated aortic stenosis. Eur. J. Cardiothorac. Surg..

[30] Hachicha Z., Dumesnil J.G., Bogaty P., Pibarot P. (2007). Paradoxical low-flow, low-gradient severe aortic stenosis despite preserved ejection fraction is associated with higher afterload and reduced survival. Circulation.

[31] Tastet L., Kwiecinski J., Pibarot P., Capoulade R., Everett R.J., Newby D.E., Shen M., Guzzetti E., Arsenault M., Bédard É. (2020). Sex-Related Differences in the Extent of Myocardial Fibrosis in Patients With Aortic Valve Stenosis. JACC Cardiovasc. Imaging.

[32] Kararigas G., Dworatzek E., Petrov G., Summer H., Schulze T.M., Baczko I., Knosalla C., Golz S., Hetzer R., Regitz-Zagrosek V. (2014). Sex dependent regulation of fibrosis and inflammation in human left ventricular remodelling under pressure overload. Eur. J. Heart Fail..

[33] Naoum C., Blanke P., Dvir D., Pibarot P., Humphries K., Webb J., Leipsic J. (2016). Clinical Outcomes and Imaging Findings in Women Undergoing TAVR. JACC Cardiovasc. Imaging.

[34] Treibel T.A., Kozor R., Fontana M., Torlasco C., Reant P., Badiani S., Espinoza M., Yap J., Diez J., Hughes A.D. (2018). Sex Dimorphism in the Myocardial Response to Aortic Stenosis. JACC Cardiovasc. Imaging.

[35] Kwak S., Singh A., Everett R.J., Treibel T.A., Lim J., Won S., Williams M.C., Loganathan K., Bing R., Craig N. (2025). Sex-Specific Association of Myocardial Fibrosis With Mortality in Patients With Aortic Stenosis. JAMA Cardiol..

[36] Zwadlo C., Schmidtmann E., Szaroszyk M., Kattih B., Froese N., Hinz H., Schmitto J.D., Widder J., Batkai S., Bähre H. (2015). Antiandrogenic therapy with finasteride attenuates cardiac hypertrophy and left ventricular dysfunction. Circulation.

[37] Chehab O., Shabani M., Varadarajan V., Wu C., E Watson K., Yeboah J., Post W.S., Ambale-Venkatesh B., A Bluemke D., Michos E.D. (2023). Endogenous sex hormone levels and myocardial fibrosis in men and postmenopausal women. JACC Adv..

[38] Greiten L.E., Holditch S.J., Arunachalam S.P., Miller V.M. (2014). Should there be sex-specific criteria for the diagnosis and treatment of heart failure?. J. Cardiovasc. Transl. Res..

[39] Shub C., Klein A.L., Zachariah P.K., Bailey K.R., Tajik A.J. (1994). Determination of left ventricular mass by echocardiography in a normal population: Effect of age and sex in addition to body size. Mayo Clin. Proc..

[40] Sickinghe A.A., Korporaal S.J.A., den Ruijter H.M., Kessler E.L. (2019). Estrogen contributions to microvascular dysfunction evolving to heart failure with preserved ejection fraction. Front. Endocrinol..

[41] Petrov G., Regitz-Zagrosek V., Lehmkuhl E., Krabatsch T., Dunkel A., Dandel M., Dworatzek E., Mahmoodzadeh S., Schubert C., Becher E. (2010). Regression of myocardial hypertrophy after aortic valve replacement: Faster in women?. Circulation.

[42] Zhou L., Shao Y., Huang Y., Yao T., Lu L.M. (2007). 17-Estradiol inhibits angiotensin II-induced collagen synthesis of cultured rat cardiac fibroblasts via modulating angiotensin II receptors. Eur. J. Pharmacol..

[43] Michail M., Davies J.E., Cameron J.D., Parker K.H., Brown A.J. (2018). Pathophysiological coronary and microcirculatory flow alterations in aortic stenosis. Nat. Rev. Cardiol..

[44] McConkey H.Z.R., Marber M., Chiribiri A., Pibarot P., Redwood S.R., Prendergast B.D. (2019). Coronary Microcirculation in Aortic Stenosis. Circ. Cardiovasc. Interv..

[45] Reynolds H.R., Bairey Merz C.N., Berry C., Samuel R., Saw J., Smilowitz N.R., de Souza A.C.D.A., Sykes R., Taqueti V.R., Wei J. (2022). Coronary Arterial Function and Disease in Women With No Obstructive Coronary Arteries. Circ. Res..

[46] Oguz D., Huntley G.D., El-Am E.A., Scott C.G., Thaden J.J., Pislaru S.V., Fabre K.L., Singh M., Greason K.L., Crestanello J.A. (2023). Impact of atrial fibrillation on outcomes in asymptomatic severe aortic stenosis: A propensity-matched analysis. Front. Cardiovasc. Med..

[47] Avakian S.D., Tarasoutchi F., Mansur A. Left ventricular mass index and prognosis of symptomatic severe aortic stenosis in women and men. Proceedings of the European Congress of Cardiology.

